# Locally correlated kinetics of post-replication DNA methylation reveals processivity and region specificity in DNA methylation maintenance

**DOI:** 10.1098/rsif.2022.0415

**Published:** 2022-10-26

**Authors:** Honglei Ren, Robert B. Taylor, Timothy L. Downing, Elizabeth L. Read

**Affiliations:** ^1^ NSF-Simons Center for Multiscale Cell Fate, University of California, Irvine, CA 92697, USA; ^2^ Department of Chemical and Biomolecular Engineering, University of California, Irvine, CA 92697, USA; ^3^ Center for Complex Biological Systems, University of California, Irvine, CA 92697, USA; ^4^ Department of Physics, University of California, Irvine, CA 92697, USA; ^5^ Department of Biomedical Engineering, University of California, Irvine, CA 92697, USA; ^6^ Department of Microbiology and Molecular Genetics, University of California, Irvine, CA 92697, USA

**Keywords:** DNA methylation, processivity, stochastic modelling

## Abstract

DNA methylation occurs predominantly on cytosine-phosphate-guanine (CpG) dinucleotides in the mammalian genome, and the methylation landscape is maintained over mitotic cell division. It has been posited that coupling of maintenance methylation activity among neighbouring CpGs is critical to stability over cellular generations; however, the mechanism is unclear. We used mathematical models and stochastic simulation to analyse data from experiments that probe genome-wide methylation of nascent DNA post-replication in cells. We find that DNA methylation maintenance rates on individual CpGs are locally correlated, and the degree of this correlation varies by genomic regional context. By using theory of protein diffusion along DNA, we show that exponential decay of methylation rate correlation with genomic distance is consistent with enzyme processivity. Our results provide quantitative evidence of genome-wide methyltransferase processivity *in vivo*. We further developed a method to disentangle different mechanistic sources of kinetic correlations. From the experimental data, we estimate that an individual methyltransferase methylates neighbour CpGs processively if they are 36 basepairs apart, on average. But other mechanisms of coupling dominate for longer inter-CpG distances. Our study demonstrates that quantitative insights into enzymatic mechanisms can be obtained from replication-associated, cell-based genome-wide measurements, by combining data-driven statistical analyses with hypothesis-driven mathematical modelling.

## 1. Introduction

DNA methylation is an important epigenetic modification that plays a critical role in development, ageing and cancer, and it is well conserved among most plants, animals and fungi [1,2]. In mammals, DNA methylation occurs predominantly in the cytosine-phosphate-guanine (CpG) dinucleotide context. Across most of the mammalian genome, CpGs occur with low frequency, except for regions called CpG islands (CGIs), which are often associated with promoters [3]. Methylated promoters are associated with transcriptional repression, pointing to a role for DNA methylation as a stable and heritable chromatin mark to programme alternative gene expression states [4,5].

The inheritance and maintenance of methylation patterns across cell cycles is important in development and throughout organismal lifespan. Methylation patterns encode information related to gene expression [6,7], differentiation [8] and genomic imprinting [9]. Failure in maintenance and transmission of such patterns can lead to aberrant gene expression, and diseases including cancer [10], developmental abnormalities and even death [11]. The classical model of DNA methylation maintenance introduced the idea that the symmetrical nature of the CpG dinucleotide provides a biomolecular structure whereby DNA methylation could be inherited across a single-CpG site by the activity of a, then posited, ‘maintenance’ methyltransferase enzyme [9,12]. The mammalian DNA methyltransferase DNMT1 was subsequently found to serve as the primary maintenance enzyme [13]. However, the classical model has been refined in a number of ways based on updated understanding of the biochemical properties and genomic activity of methyltransferases (reviewed in [14]). For instance, a wealth of evidence suggests that the efficiency and specificity of methylation enzymes are not sufficient to support the observed high fidelity of maintenance, within the classical, independent-site model [15–19].

Interdependence, or coupling, of maintenance methylation activities imparted on CpGs located within close proximity can provide some reconciliation between the known biochemistry of methylation reactions and the observed stability of the genomic methylation landscape. Recent findings of preferential recruitment of DNMT1 [20], and faster maintenance methylation rates [21], at sites with more neighbouring hemimethylated CpGs are clearly at odds with an independent-CpG-site model. CpG interdependence has been suggested to occur via various molecular mechanisms, including DNMT1 processivity [22–24] (in which an enzyme can methylate multiple neighbouring CpGs on nascent DNA sequentially) and cooperative interactions, e.g. with UHRF1, which localizes, and in turn helps recruit DNMT1 molecules, to hemimethylated CpGs [25,26]. Mathematical modelling has also suggested the importance of CpG interdependence, also called collaboration, both in maintenance and de novo methylation, for long-term collective stability of methylated and unmethylated genomic regions [19,27–31].

A quantitative and mechanistic understanding of CpG interdependence during maintenance methylation *in vivo* is lacking. The genomic lengthscales over which CpG coupling occurs are not well understood. It is not yet known to what extent processivity, versus other mechanisms of CpG interdependence, influences dynamics of maintenance methylation. Nor is it yet well understood how local genomic context influences these mechanisms *in vivo*. In this study, we address these questions by elucidating CpG-coupled-dynamics in maintenance methylation by use of statistical inference, bioinformatics and stochastic modelling. We leverage experiments that measured methylation status of nascent-strand CpGs across post-replication timescales, genome-wide [32]. From these data, we infer how the rates with which individual CpGs acquire methylation, post-replication, are correlated on nearby sites in different regional contexts. By using stochastic models and theory of proteins diffusing along DNA, we demonstrate that the rate correlation as a function of genomic distance provides a mechanistic fingerprint for post-replication enzymatic processes. Our method provides a novel way to infer lengthscales of linear diffusion of DNA-binding proteins, and it provides the first direct evidence for genome-wide methyltransferase processivity in cells. Comparing simulations with data allows us to extract quantitative insights from data, including the relative strengths of processive versus non-processive coupling mechanisms in different genomic regions, and the length of processive steps.

## 2. Results

### 2.1. Overview of method

The methodology of this article can be summarized as follows. We reanalysed data from whole genome bisulfite sequencing (WGBS) [33] and replication-associated bisulfite sequencing (Repli-BS) [32] in human embryonic stem cells (hESCs) using a combination of data-driven statistical inference and hypothesis-driven stochastic modelling. First, maximum likelihood estimation (MLE) was used to infer per-CpG post-replication remethylation rates from Repli-BS experiments, following our previously developed method [34]. We analysed the correlation of these data-inferred rates on nearby CpGs in different genomic contexts, such as enhancer, promoter, etc., to study regional differences in maintenance kinetics. Next, we studied the association of the strength of nearby-CpG remethylation rate correlations with other local genomic/epigenomic features. To aid interpretation of the experiment-derived correlation functions and their regional differences and associations, we developed region-specific stochastic models of post-replication DNA methylation maintenance kinetics. By using these models, we generated simulated bisulfite sequencing datasets under different mechanistic hypotheses, and we compared the resultant *in silico*-generated rate correlation functions with those of the experimental data, focusing both on qualitative and quantitative features, such as shape and lengthscale. Combining theory with stochastic simulation, we developed a method to separate processive and non-processive contributions to the experiment-derived rate correlations.

### 2.2. DNA methylation *rate* and *state* on neighbouring CpGs are correlated with different extents

An analysis of cytosine methylation within newly replicated DNA over time (via Repli-BS) revealed that some genomic loci exhibit a pronounced lag in methylation maintenance [32]. In our previous work, we developed a statistical inference procedure to obtain single-CpG post-replication maintenance methylation rates (here denoted ‘remethylation rates’) from Repli-BS data [34], which further supported the variability of remethylation kinetics across the genome. Other recent studies found variable kinetics of maintenance methylation in different genomic contexts [21] and of combined methylating/demethylating reactions [35]. However, these studies did not investigate whether or how methylation rates vary locally within regions or among neighbouring clusters of CpGs.

In this article, we focus on local correlation of methylation kinetics, obtained from Repli-BS data, and analyse the data-derived correlation using biophysical models of enzymatic methylation reactions. The data-inferred kinetic parameters quantify the rate of accumulation of methylation at each CpG site across hESCs in the measurement set over the experimental timecourse of 0–16 h post-replication. We correlate single-CpG remethylation rate constants (denoted *k*_*i*_, for the rate at the *i*th CpG, obtained by MLE, see §4) on pairs of CpGs, as a function of the genomic distance between them. As such, this post-replication methylation *rate correlation* quantifies the extent to which CpG pairs experience similar (fast or slow) kinetics.

DNA methylation levels (also from here on denoted methylation ‘states’, or the fraction of cells exhibiting methylation at an individual CpG site) on neighbouring CpGs are also correlated [30,36]. This methylation *state correlation*, as a function of genomic distance in basepairs, reflects information such as the size of persistently methylated (or unmethylated) domains. As such, it reflects the generally static methylation landscape in a given cell type.

**Figure 1.**
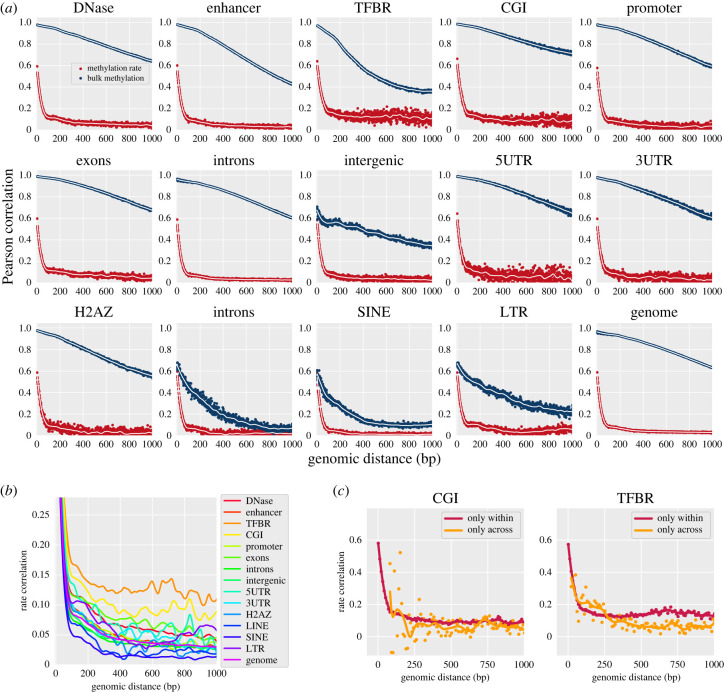
Methylation states and post-replication remethylation rates are correlated on neighbouring CpGs, and correlation varies with genomic regional context. (*a*) Correlation of remethylation rates (red, *y*-axis) and correlation of per cent methylation (i.e. bulk methylation state, blue, *y*-axis) of nearby CpGs at given genomic distances (*x*-axis), separated in panels by genomic regional context. Each scatter point at distance *d* represents the correlation coefficient of rate pairs {*k*_*X*_, *k*_*Y*_} derived from all CpG pairs in the region with intervening distance *d*. The remethylation rates for individual CpG sites were inferred from experimental Repli-BS-seq data, and the bulk methylation was obtained from separate WGBS data from the same cell line (HUES64). Dots: raw rate correlation in base pair resolution, lines: smoothed correlation curve by LOESS (locally estimated scatterplot smoothing, [37]) with 100 bp span. (*b*) Correlation of remethylation rates with curves from different genomic regional contexts overlapped and *y*-axis zoomed. Only the LOESS-smoothed curves are shown for clarity. (*c*) Rate correlation functions computed retaining only pairs of sites identified either as within the same localized region (red) or not within the same localized region (orange).

We reasoned that correlation of post-replication methylation rates on individual CpGs could reveal details of enzyme kinetics in cells, and thereby yield additional information beyond that contained in methylation state correlation. To this end, we first computed correlation from the two different data modalities ((i) remethylation rate derived from Repli-BS and (ii) methylation state derived from WGBS) to relatively short (less than 1 kilobasepair) distances. We compared them across different genomic contexts, by filtering CpG pairs by genomic context in addition to their inter-CpG distance (figure 1*a*). Genomic regional contexts were based on genomic annotations acquired from the UCSC genome table browser and included features such as 3’UTRs, enhancers, etc. (see §4). Note that a given CpG can be located within multiple regional contexts, e.g. CGIs are often overlapping with promoters.

We observe both common and distinct features in the correlation functions, when comparing different genomic regional contexts for both data modalities (i.e. rate correlations and state correlations). Common to all regions and to the genomic average with no region-filtering, remethylation rate correlation decreases rapidly with distance until approximately 100 bp (red curves in figure 1*a*). After 100 bp, a slower decay is evident in all regions. However, this steep decrease in correlation, and rapid change in decay is not observed in correlation of methylation state (navy curves), which generally shows a slower (persisting to greater than 1 kb), smooth decay, albeit with wide variation in decay lengths between regions. The existence of such a pronounced difference between methylation state and rate correlation, persistent in all genomic regions, suggests that the methylation rate correlations are not determined by the methylation landscape itself and raises the question of what dynamic processes determine rate correlations.

### 2.3. Post-replication remethylation rates are correlated on neighbouring CpGs, and correlation varies with genomic regional context

Some features of the correlation functions vary between the genomic regions studied. We quantify the average magnitude of short-range correlation by the mean correlation to 1 kbp. These magnitudes are highly variable across genomic contexts and across modalities. Genome-wide, the magnitude of rate correlation is low (0.06) compared with that of state correlation (0.82). Across regions, CGIs show the highest magnitude of state correlation (0.85), while transcription factor binding regions (TFBRs) show the highest magnitude of rate correlation (0.15). By contrast, both the lowest average magnitude of state correlation and of rate correlation are found within SINE (short interspersed nuclear element) regions with 0.18 and 0.035 correlation, respectively. We hereafter refer to these differences in remethylation rate and state correlations according to genomic context as ‘genomic region specificity’.

In general, the results for methylation state indicates that methylation on CpGs within 1 kb are highly correlated genome wide. Exceptions are within SINEs and LINEs (long interspersed nuclear elements), where state correlation has significantly decayed by 1 kbp.

Although the methylation rate correlations show an apparently uniform sub-100 bp decay across regions, differences are visible in their longer-lengthscale (greater than 100 bp) decay profiles (figure 1*b*). In particular, CGIs and TFBRs show the strongest long-distance correlation, persisting near or above 0.1 past 1 kbp. We further separated the analysed CpGs from CGIs and TFBRs into ‘within’ and ‘across’ region pairs (figure 1*c*). ‘Within’ correlation is computed for CpGs that are within one contiguous region (as defined by the filtering protocol), whereas ‘across’ correlation only retains pairs of sites that are assigned to non-contiguous regions. The resultant rate correlation functions indicate generally stronger contribution of within- versus across-region correlation (note that the number of datapoints for ‘across’ correlations is lower for short distances, since nearby CpGs are more likely to be in contiguous regions). Size distributions of all studied genomic regions are shown in electronic supplementary material, figure S1.

In all, these results indicate different magnitudes and decay lengths of inter-CpG coupling in maintenance methylation. In particular, they point to more pronounced CpG collectivity of maintenance kinetics in contiguous CGIs and TFBRs. That is, neighbouring CpGs in CGIs and TFBRs tend to have more correlated remethylation rates and thus more similar methylation across post-replication time, compared with other regions. However, all regions showed correlated rates with some degree.

We note several additional features in the correlation functions, including apparent periodicity (e.g. in state correlation, intergenic region, figure 1*a*) and shoulders or local peaks (e.g. in several of the rate correlation curves in figure 1*b*). Some of these features appear consistent with nucleosome positioning, which could indicate coupling between maintenance methylation processes and/or the methylation landscape with nucleosomes. A detailed investigation of all of these features is outside the scope of this work; we focus instead on the broad features of the methylation rate correlation, namely, the sub-100 bp rapid decay and region-specific, less than 100 bp slower decay.

### 2.4. Regional correlation of DNA methylation maintenance kinetics is increased with CpG density and chromatin accessibility, and decreased with higher bulk methylation levels

**Figure 2.**
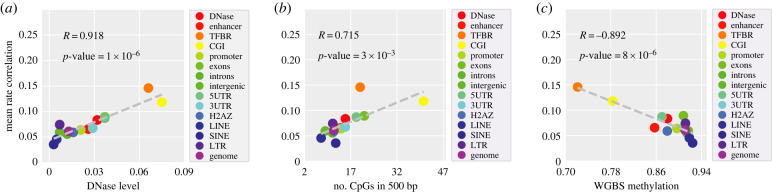
DNA methylation maintenance rates show higher local correlation in genomic regions with higher chromatin accessibility, CpG density and lower bulk methylation levels. Mean remethylation rate correlation by region, plotted versus other quantified, localized genomic measurements from independent measurements: (*a*) magnitude of mean remethylation rate correlation (equal to the average over rate correlation for all inter-CpG distance less than 1 kbp (i.e. integers from 2 to 1000) in the given region) versus mean regional chromatin accessibility, as quantified by DNase level; (*b*) versus mean local CpG density (mean number of neighbouring CpGs within a 500 bp window); (*c*) versus mean WGBS (‘bulk’) methylation level. Note that the datasets are site matched, so the analysis is restricted to sites that tend to have intermediate to high methylation, since these are the sites for which remethylation rates are available.

To investigate the factors associated with the observed region specificity, the association between remethylation rate correlation and other local genomic characteristics were examined. For each individual CpG in the dataset, a measure of the local chromatin accessibility is collected from DNaseI hypersensitivity data in ENCODE/OpenChrom (see §4). The local CpG density surrounding a given site is calculated by the number of neighbouring CpGs within a 500 bp window. ‘Bulk’ methylation refers to measurement of DNA methylation in human embryonic stem cells (HUES64) measured by WGBS. We plotted the magnitude of the remethylation rate correlation (0–1000 bp) for each region versus average chromatin accessibility (e.g. DNAse hypersensitivity enrichment) (figure 2*a*), local CpG density (figure 2*b*) and WGBS (‘bulk’) methylation percentage (figure 2*c*). We calculated the Pearson correlation coefficients (and associated *p*-values) between the mean rate correlations and three factors using the ‘stats.pearsonr’ in the Scipy package [38]. Linear correlation was found between the magnitude of remethylation rate correlation and these three factors. We found that the rate correlation is positively correlated with DNase level and CpG density (albeit weakly), while it is inversely correlated with bulk methylation level. These results suggest that the genomic regional differences observed in rate correlation in figure 1 may be driven globally by variation in chromatin accessibility, CpG density and background methylation landscape.

### 2.5. Local methylation correlation varies across post-replication time and shows persistent region specificity

**Figure 3.**
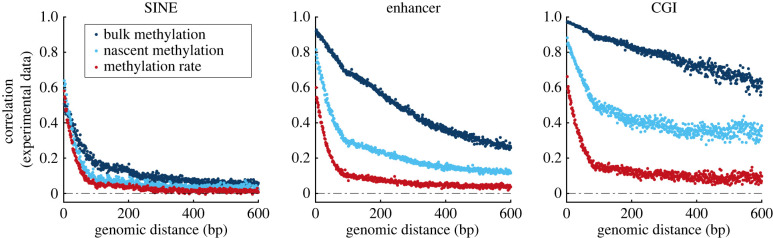
DNA methylation correlation increases over post-replication time and shows persistent region specificity across different data modalities. Data from three representative regions and three methods of extracting methylation correlation; all three curves in each panel are derived from the same set of CpGs. Dark blue: correlation of methylation state of neighbouring CpGs from whole genome bisulfite sequencing experiments (WGBS, ‘bulk’). Light blue: correlation of methylation state of neighbouring CpGs from WGBS on nascent DNA from Repli-BS-seq experiments (i.e. less than 1 h post-replication) (‘nascent’). Red: correlation of remethylation rates inferred from Repli-BS-seq experiments. Data for rate correlation (red) is identical to that of figure 1*a*. Data for bulk methylation (WGBS) is also derived from the same dataset as figure 1*a*, but filtered to retain only those CpG sites for which a rate was available (determined by per-site read-depths, see §4). Note that the nascent (light blue) and rate (red) curves are not from independent datasets, since the nascent data are a subset of the total Repli-BS-seq dataset from which the rates were inferred. Blue Pearson correlation functions are computed from raw data, whereas the red correlation function is computed on inferred rate parameters from the MLE pipeline.

To investigate the origins and regional differences of methylation correlation, we focus on three representative genomic regions characterized by high (CGI), medium (enhancer) and low (SINE) methylation state correlation. In figure 3, we plot three types of correlation functions for these three regions: in addition to bulk WGBS correlation and rate correlation, we also plot correlation of methylation state in ‘nascent’ DNA, which contains a subset of measurements from the full temporal Repli-BS dataset, corresponding to the 0-hour timepoint of the original pulse-chase experiment[32]. Thus, ‘nascent’ here refers to methylation readout less than or equal to 1 h post-replication.

Note that the red rate and dark blue state correlations are derived from the same data as shown in figure 1, but in contrast to figure 1, the curves in each panel of figure 3 are site matched. In practice, remethylation rates are available for only a subset of CpGs compared with WGBS measurements. This is because the rate constant is undefined where no methylation is measured and is often unidentifiable when methylation is very low. Thus, there is a significant overlap between the sites for which rates are not available and sites for which WGBS percentages are 0 or near 0. We reasoned that some of the methylation state correlation in figure 1 could arise from the bimodal nature of methylated and unmethylated regions. For a more direct comparison with rate correlation on a site-specific basis, we thus filtered to a common set of sites, which in practice retains mostly sites with intermediate to high methylation in WGBS. After filtering to these common sites, we indeed observe some decrease in state correlation (dark blue curves in figure 1*a* versus figure 3), but rate correlation remained persistently lower than state correlation in all regions.

We observe that methylation state correlation in nascent DNA is generally lower than that in bulk DNA, suggesting that state correlation increases over post-replication time. (Bulk WGBS measurements reflect temporal variability from cells in various stages of the cell cycle and from differences in replication timing across the genome (meaning it largely contains matured DNA strands), whereas the nascent data in principle captures reads within 1 h post-replication [32]). In all studied regions, the nascent methylation correlation is intermediate between rate and bulk state correlation (with the exception of very short distances in SINEs).

The trend in genomic region specificity of methylation correlation is persistent across the three types of correlation functions. This in turn suggests that the region specificity is persistent across post-replication time. For example, CGIs consistently show the highest correlation compared with the other regions in rate, bulk methylation, and nascent methylation. Conversely, SINE consistently shows the lowest correlation. These results suggest that the processes that govern coupling (or interdependence) of methylation among neighbouring CpGs differ depending on the genomic regional context, and that these processes are region specific already at early post-replication timepoints. In addition, the distinct shapes and magnitudes of the CpG–neighbour correlations across time suggest that different processes control CpG–neighbour interactions at early versus late post-replication times.

We hypothesize that the three correlation modalities can be interpreted as follows: rate correlation reflects the dynamic mechanisms of maintenance methylation, thus shedding light on early post-replication time processes. In contrast, bulk methylation state correlation largely reflects the steady-state methylation landscape, i.e. reflecting the balance among methyl-reading/writing/erasing processes operating across post-replication time to regulate the methylation landscape, but largely reflecting the stable methylation landscape of a given cell type. Nascent methylation state reflects a mixture of the two, as the experiment ‘captures’ CpGs in transit between their state immediately (up to 1 h) post-replication and steady state. In the following sections, we test this hypothesis by use of computer simulations and model-guided data analysis.

### 2.6. Region-specific stochastic simulations of post-replication maintenance methylation

**Figure 4.**
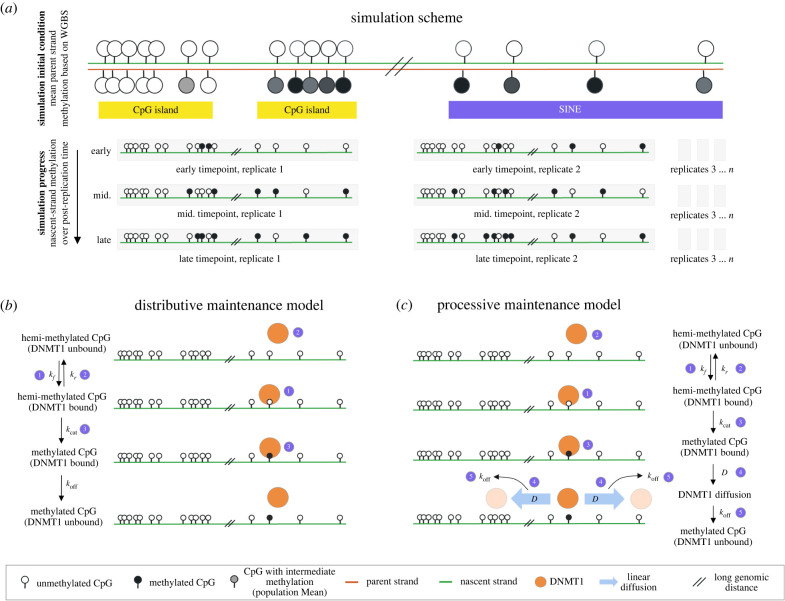
Schematic of stochastic simulation of DNA methylation maintenance in different genomic regions, according to either distributive or processive mechanisms. (*a*) Each simulation models a strand of *N* CpGs (*N* =25 000–75 000), where the CpG positions and the parent-strand methylation at the initial condition are taken from WGBS measurements in a given genomic region (e.g. CGI or SINE). Multiple-strand replicates are simulated over time. Immediate-post-replication DNA is assumed to be unmethylated on the nascent strand at all sites, and the binary methylation status of the parent-strand sites are sampled probabilistically from the input methylation landscape (mean methylation level in WGBS). During simulations, DNMT1 targets hemimethylated CpGs for methylation, according to either a distributive (*b*) or processive (*c*) mechanism. The distributive mechanism assumes that the enzyme binds to each hemi-methylated CpG independently. After catalysing methylation, the enzyme immediately unbinds from DNA (we assume *k*_off_ ≫ *k*_cat_, such that methylation and unbinding are treated as a single reaction). To reach a subsequent CpG, the enzyme must independently rebind with rate *k*_*f*_. The processive mechanism assumes that, after catalysing methylation at a CpG, DNMT1 can remain bound to DNA and reach nearby hemimethylated CpGs by linear diffusion along DNA.

To gain further biological insight from the experiment-derived methylation correlation functions, we perform region-specific stochastic simulations of maintenance methylation (figure 4) and use these simulations to generate synthetic data analogous to the various experimental bisulfite sequencing data modalities. From these synthetic data, we compute regional correlation functions and compare with those derived from experiments. Briefly (see §4), the simulations track nascent-strand methylation status of stretches of sequentially positioned CpGs, numbering of the order of tens of thousands. In contrast to mathematical models that treat the interplay of de novo, maintenance and demethylating reactions, e.g. [19], we apply minimal models of single-site-resolution DNMT1-mediated methylation on post-replication timescales. Each stochastic simulation tracks the binary (methylated or not methylated) status of the ‘nascent-strand’ CpGs. At the start of the simulation (representing exactly time 0 with respect to DNA replication at that site, i.e. the time of nucleotide addition), all nascent CpGs are assumed to be unmethylated. The presence or absence of methylation on cytosine bases on the opposing parental strand at time 0 is determined probabilistically from a data-derived regional methylation landscape that acts as the simulation input. If the parental cytosine is methylated at time 0, then the CpG is considered hemimethylated and the nascent cytosine is assumed to be a target for DNMT1-catalysed methylation, and it will acquire methylation stochastically at some post-replication timepoint, according to the chemical reaction kinetics encoded in the model. If the parental cytosine is unmethylated at time 0, then DNMT1 does not target the nascent cytosine for methylation, and the site will remain fully unmethylated. In this way, the model tracks only unidirectional maintenance methylation and does not include active demethylation reactions. It also does not account for any de novo methylation activity. The simulation tracks post-replication timescales (following experiments, to approximately 16 h), up to but not including subsequent replication events.

Region specificity is encoded at the start of the simulation in two ways: (i) the CpG positions and (ii) the local methylation landscape, meaning: the probability of the nascent CpG to be a target for DNTM1-catalysed methyl addition due to the presence of methylation on the parental strand CpG dinucleotide. Both of these quantities are derived from experimental WGBS data with a regional filter to retain only CpGs in the desired region. Thus, in simulating a CGI region, the *i*th simulated CpG (*i* ∈ [1..*N*]) has a genomic position *x*_*i*_ and a probability *f*_*i*_ to be targeted for methylation. We obtain both *x*_*i*_ and *f*_*i*_ from WGBS data from hESCs, where *x*_*i*_ is the integer site ID for the cytosine (which is identified as being located within a CGI) and *f*_*i*_ ∈ [0, 1] is taken to be equal to the measured methylation fraction at that site. For example, if a given CpG in the dataset has a WGBS-measured methylation fraction of 0.8, then the model assigns the parental cytosine to be methylated at the start of the simulation with a probability equal to 0.8. Strands are simulated in replicate. With sufficient replicates, the simulation eventually recapitulates the experiment-derived input methylation landscape, if it is run for a period of time that is sufficiently long. That is, the simulation assumes perfect recapitulation of parent-strand methylation by DNMT1 eventually, though the time at which each hemimethylated CpG gains nascent-strand methylation is stochastic. In practice, the number of replicates and sampled timepoints are chosen to match those of the experimental data (see §4). Note that the WGBS data-derived landscape probably reflects some degree of replication-associated temporal variability [32], rather than a true steady state. Nevertheless, the use of the WGBS background methylation landscape as the simulation input allows us to encode realistic region-specific differences in CpG densities and qualitative differences in bulk methylation levels.

### 2.7. Rate correlation provides a mechanistic fingerprint for enzyme kinetics

**Figure 5.**
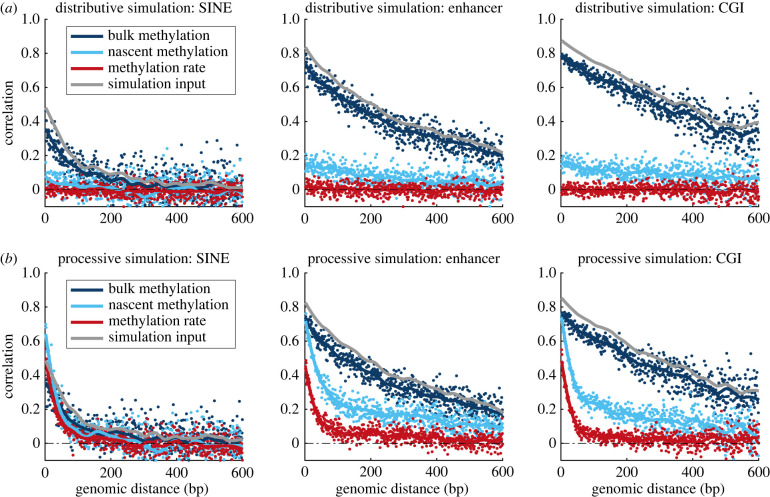
In simulations, rate correlation but not state correlation depends on enzymatic mechanism; only processive model displays non-zero rate correlation. Simulated correlation functions for three representative regions (SINE, enhancer, CGI) and using the distributive (*a*) or processive (*b*) mechanism. Simulations are performed using experimental regional methylation landscapes for SINE, enhancer and CGI as initial condition inputs (grey curves). The simulations provide synthetic data corresponding to each of the three experimental data modalities, as shown in figure 3. Synthetic data are processed in the same way as the experimental data to compute the inferred remethylation rates and Pearson correlation functions. Both models recapitulate temporal trends in rate correlation seen in experiments, figure 3 (correlation in rate < nascent state < bulk state). Only the processive model captures non-zero rate correlation sub-100 bp; neither model captures low, persistent correlation greater than 100 bp observed in figure 3.

We use simulations to generate synthetic data mimicking the various bisulfite sequencing datatypes (rates from Repli-BS, nascent methylation from Repli-BS, and bulk methylation state from WGBS). We then compute the correlation functions for the synthetic data. Figure 5 shows simulation-derived correlation functions for three representative genomic regions from chromosome 1 for two mechanistic models termed distributive and processive. Briefly, DNMT1 binds to nascent CpGs and catalyses the addition of a methyl group. In the distributive model (figure 4*b*), the enzyme unbinds after the catalytic step and must independently rebind to an available hemimethylated CpG to catalyse a subsequent methyl addition. In the processive model (figure 4*c*), the enzyme can remain bound to DNA after methylating a CpG and can travel along DNA (via a one-dimensional diffusive random walk) to reach neighbouring hemi-methylated CpGs and again catalyse methylation. The random walk occurs with one-dimensional diffusion coefficient, *D*, and the enzyme potentially unbinds before reaching its target with rate *k*_off_. The kinetic parameters for both models are given in the electronic supplementary material.

In both distributive and processive mechanisms, the simulated bulk methylation correlation approaches the input methylation landscape in the three regions. This is expected because the input landscape dictates parental strand methylation in the model, and the model assumes that as time progresses, the nascent-strand methylation will ultimately match the parental strand input (i.e. it assumes perfect maintenance in the long-time limit). In contrast, the shapes of remethylation rate correlation are distinct from those of the input methylation landscape and are dependent on the model mechanism. In all simulated regions, nascent correlation is intermediate between bulk state and rate, in agreement with experiments. The distributive model produces no correlation in remethylation rate, in agreement with our previous results [34]. In contrast, the processive model produces a non-zero rate correlation that appears to qualitatively reproduce the experimentally observed rapid decay of correlation with genomic distance up to approximately 100 bp. Neither model reproduces the low but persistent correlation visible at distances greater than 100 bp in CGI and enhancer in figure 3.

The simulated correlation functions provide a possible explanation for the discrepancy between methylation state and rate correlations observed from experiments (figures 1 and 3). Namely, they suggest that the methylation rate correlation shape is dictated by enzymatic maintenance mechanisms, independent of the background methylation landscape. That is, simulations support that mechanistic insights on maintenance methylation can be derived using the rate correlation, since it effectively separates correlations introduced by early post-replication time processes from those operating at longer timescales (and which dominate the bulk WGBS correlation). Thus, the rate correlation is a useful new quantity, which can be used to distinguish between hypothesized mechanisms. In the processive model, the rapid drop-off of rate correlation is due to the enzyme’s intrinsically limited capability to process along DNA over long lengthscales. As such, a pair of CpGs can be strongly correlated in their methylation state (as dictated by parent-strand methylation, and thus reflective of the correlated methylation landscape), while showing low correlation of their kinetic rates, if their distance is outside the enzyme’s processivity range. However, the simulations also demonstrate that, while the processive mechanism partially recapitulates experiment-derived rate correlations, it cannot explain the longer-distance, slow rate correlation decay in CGI.

### 2.8. Rate correlation in processive model decays exponentially, dependent on one-dimensional diffusion and unbinding rates

We used simulations and theoretical modelling to determine whether quantitative insights could be derived from the experimental rate correlations. We first investigated how rate correlations arising from the processive mechanism depend on model parameters. In an idealized theoretical model, we find that the rate correlations are related to the diffusion constant *D* and the unbinding rate, *k*_off_, as follows:

2.1 $$\mathrm{Corr}(X,Y|d)\propto e^{-d\sqrt{k_{\mathrm{off}}/D}},$$

where *X* and *Y* are pairs of remethylation rates on sites that are distance *d* apart (along the DNA strand). See the electronic supplementary material for justification of equation (2.1).

We find a good match between the aforementioned analytical theory and the stochastic simulations (electronic supplementary material, figure S2). We performed regional simulations for varying values of *D*/*k*_off_ and then processed the simulated data through the MLE inference pipeline and computed the correlation function. We then fit the correlation functions to a single-exponential decay and observe a good match between the fitted decay constant and the theoretically predicted value of $\sqrt{k_{\mathrm{off}}/D}$. These results demonstrate that the predicted exponential correlation holds, despite potentially complicating factors in the stochastic model (e.g. the finite time required for diffusion, many CpG sites and multiple enzymes acting simultaneously on one strand, etc.), and uncertainty introduced by the MLE-fitting pipeline. The simulations also predicted that the exponential decay length was not strongly affected by the length of measured reads in the Repli-BS experiments (electronic supplementary material, figure S3), though some spurious correlation arising from read length was observed *in silico*. All in all, these results support the finding that processivity, in a linear diffusion model, is consistent with exponential decay of rate correlations obtained from Repli-BS and that the decay constant can be interpreted as $\sqrt{k_{\mathrm{off}}/D}$.

### 2.9. Mathematical model for diffusion-based and region-based components of methylation rate correlation

The processive model can explain exponential decay of rate correlation, but cannot by itself explain the significant correlation observed past 100 bp in strongly correlated regions such as CGI (figure 3). Nor can it explain the association of this correlation to other regional genomic characteristics (bulk methylation levels, CpG density and chromatin accessibility). On the basis of the observations in figure 2, we reasoned that this additional rate correlation can be understood to result from correlated methylation times on neighbouring sites due to a variety of regional features that we describe as follows:

2.2 $${\mathrm{Corr}}_{\mathrm{region}}(X,Y|d)=\phi (\mathrm{features}(d)),$$

and refer to as ‘region-based correlation’. That is, this component of the correlation function can be expressed as a function *ϕ*, which depends somehow on local features, e.g. related to the chromatin landscape. These features themselves held some distance-dependent correlation. Consideration of additional correlating factors (beyond diffusion) equates to a revision of the idealized theoretical model above. Now, in event 1, if the neighbouring site is not reached by diffusion, but rather the two sites are methylated in separate events, their times *τ*_*i*_ and *τ*_*j*_ are nevertheless correlated in a way that depends not only on their distance apart but also on various other features of their location within the genome. We do not propose a mechanistic model for this additional correlation, but label it as *ϕ*, and estimate it for each region, based on the data. Given these two contributions, which we term diffusion dependent (denoted *θ*) and region dependent (*ϕ*), the total correlation from both contributions is given by

**Figure 6.**
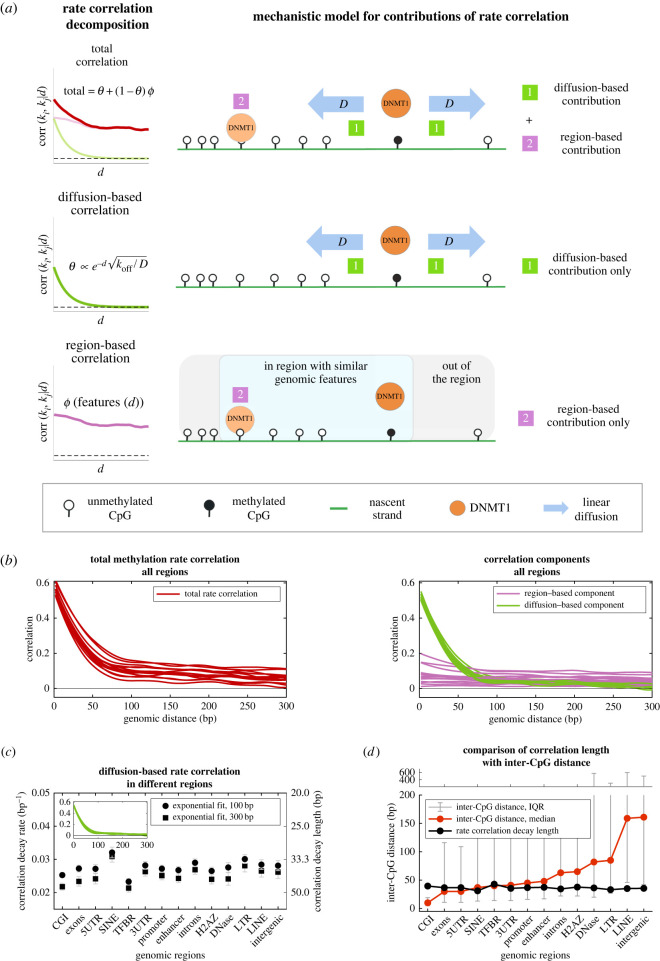
Decomposition of experimental rate correlation into processive and non-processive components allows estimation of DNMT1 diffusion parameters from data. (*a*) Mathematical model of mechanistic contributions to rate correlation predicts that the total rate correlation (red curve) arises from two types of processes, termed diffusion-based (i.e. processive) and region-based (i.e. non-processive). The diffusion-based component (green curve) is due to an individual enzyme at CpG_*i*_ reaching nearby target CpG_*j*_ by linear diffusion and shows exponential decay with genomic distance *d*. The region-based component (pink curve) is attributed to any other sources of correlation in remethylation rates, e.g. when two different enzyme molecules reach their targets (CpG_*i*_ and CpG_*j*_) with correlated arrival times, and does not have an analytic expression. (*b*) Rate correlation of all regions from experimental data is plotted in red in left panel (same red curves as figure 1*a*), and the corresponding decomposed components (see §4) are plotted in the right panel, with diffusion-based components in green and region-based components in pink. (*c*) Single-exponential fit decay constants for the green curves (*θ*), using either a 100 or 300 bp fitting window. The curves are well fit in each region by a single-exponential decay function, supporting attribution of this correlation component to the processive mechanism (i.e. to diffusion). The decay constants are relatively uniform across regions, supporting common diffusive dynamics across the genome. The average decay constant 0.028 bp^−1^ provides an estimate of $\sqrt{k_{\mathrm{off}}/D}$ and corresponds to an average distance travelled by DNMT1 to a nearby target CpG of 36 basepairs (values from 100 bp window). (*d*) Comparison of the inter-CpG distances and extracted rate correlation decay length (from *θ*) of each genomic region, showing that the exponential correlation decay length is insensitive to inter-CpG distance.

2.3 TotalCorr(X,Y|d)=θ+(1−θ)ϕ,

where the aforementioned equation can be understood by probabilistic arguments, i.e. the probability that a neighbour is reached by diffusion is *θ* (given by equation (2.1)); additional correlation *ϕ* is only present when the neighbour is *not* reached by diffusion, with probability 1 − *θ*. This mathematical decomposition of the rate correlation is shown schematically in figure 6*a*. The model of equations (2.1), (2.2) and (2.3) makes a prediction: if *ϕ* (the component of correlation due to genomic regional features) can be estimated from the data, then the remaining correlation (*θ*) should decay exponentially with distance and should not depend on local genomic context.

### 2.10. Separation of experimental remethylation rate correlation functions into diffusion-dependent and region-dependent components quantifies genome-wide processivity of DNMT1 *in vivo*

We developed an approach to estimate *ϕ* as follows. The total experiment-derived correlation functions are computed from a list of cytosine positions and their inferred remethylation rates. Denote $x_{n}^{m}$ as the position of site *n*, identified as belonging to region *m* (where *m* is 1 of the 14 regions of figure 1, and *n* ∈ [1, *N*] given that data are available for *N* sites in a region of interest). Denote the remethylation rate as $k_{n}^{m}$, giving a list of pairs {*x*_*n*_, *k*_*n*_}_*m*_ for each region, from which the correlation is computed. We also have additional genomic feature data: let $q_{n}^{m}$, $r_{n}^{m}$ and $s_{n}^{m}$ denote the measured bulk methylation level, the local CpG density and the chromatin accessibility acquired from independent datasets (see §4). We then perform unsupervised k-means clustering on the features {*q*, *r*, *s*}_*m*_ to obtain clusters of sites that are (i) previously assigned as belonging to the same type of genomic region *m* and (ii) share more fine-grained similarity in terms of their bulk methylation level, CpG density and chromatin accessibility. We then randomly shuffle the nucleotide positions within each subcluster and denote these shuffled positions as ${\tilde{x}}_{n}$. We now have a new list, ${\left\{{\tilde{x}}_{n},k_{n}\right\}}_{m}$, where the true nucleotide positions have been randomized, but their reassigned position is still similar to the true position in terms of the features {*q*, *r*, *s*}_*m*_.

We recalculate the correlation functions for each of the regions with the new lists ${\left\{{\tilde{x}}_{n},k_{n}\right\}}_{m}$. We label this new correlation function as the region-dependent component *ϕ*, reasoning that it captures rate correlation that can be attributed to the regional features, according to the model of equation (2.2). We then extract *θ* from the total correlation using equation (2.3). The results of this decomposition are plotted in figure 6*b* (a more detailed view in regions CGI, enhancer and SINE is shown in electronic supplementary material, figure S4). We find that *ϕ* is more variable between different genomic regions, as compared with the component *θ*, which appears nearly uniform across genomic regions and in all regions shows rapid decay within approximately 100 bp. We find that *θ* is generally well fit by a single-exponential decay and the fitted decay constants are similar across genomic regions, with a mean decay constant of 0.028 (ranging from 0.023 to 0.032, in TFBR and SINE, respectively), corresponding to a mean decay length of 36 basepairs (31–43 bp, respectively), figure 6*c*. Importantly, this decomposition approach does not impose any *a priori* assumptions on the functional form of *θ*. The results confirm our hypothesis that after removal of the correlation component dependent on regional features of the chromatin landscape, the remaining correlation decays exponentially with distance.

Our model predicts that the fitted exponential decay constant should be reflective of parameters of enzyme diffusion, namely, equal to $\sqrt{k_{\mathrm{off}}/D}$. Thus, the model predicts that the decay constant is insensitive to other factors, such as variability of inter-CpG spacing. We confirm this in the experiment-derived correlation functions, by comparing the fitted decay lengths in each region to the inter-CpG distance distributions in each region (figure 6*d*). Despite the significant differences in CpG density in the different regions, the decay lengths are generally constant, i.e. the median inter-CpG distance is 10 and 161 bp in CGI versus intergenic regions, with corresponding fitted decay lengths of 39.6 and 35.6 bp, respectively.

Note that the exponential fit is not perfect, as evident by slight discrepancies in fit constants obtained for different window sizes. This discrepancy is due to low, persistent, non-zero longer-range correlation visible in *θ* in some regions (electronic supplementary material, figure S4), which we attribute to our model of *ϕ*, which was based only on three genomic features, and thus probably did not fully account for all region-based correlation. We therefore report fit constants from the 100 bp window, to estimate the short-range decay while minimizing contamination from long-range residual correlation. However, the quantitative impact of different fit window sizes on estimated constants is relatively minor, as shown in figure 6*c*.

All in all, these findings support that *θ* reflects the diffusion-based contribution to the correlation function because (i) it is the explicitly distance-dependent part that remains after removing correlation attributed to the other features {*q*, *r*, *s*}_*m*_, (ii) it decays exponentially, and (iii) it is uniform across the genome, consistent with processivity being an inherent property of DNMT1’s mode of action, and thus uniform across genomic regions. If *θ* is interpreted as reflecting processivity of the enzyme according to a one-dimensional diffusion model, then diffusion parameters can be obtained from the experiment-derived correlation function. We thus estimate *D*/*k*_off_ to be 1300 bp^2^ and the length of a processive step of DNMT1 to be 36 bp, on average, across the genome.

## 3. Discussion

### 3.1. Summary of key results and methodological contribution

We have analysed correlations among CpG sites in the genome obtained from estimated kinetics of post-replication DNA methylation and from WGBS in hESCs. We find that post-replication remethylation rates on nearby CpGs are correlated, and the nature of this correlation can shed light on molecular mechanisms of maintenance methylation, when analysed in conjunction with stochastic simulation and mathematical models. We summarize our key results as follows: (i) methylation rate correlation is a new genomic quantity, which contains information distinct from that contained in methylation state correlation. In particular, the rate correlation reveals mechanistic information on enzymatic processes. (ii) Some, but not all, of the rate correlation observed on nearby CpGs decays exponentially with genomic distance and is consistent with processive activity of DNMT1 according to a linear diffusion model. Our analysis indicates an average distance of 36 bp between nearby CpGs that are methylated processively after DNA replication. (iii) In addition to evidence of processivity, we also discovered additional correlation, not consistent with a processive model, that is dependent on the genomic regional context. Among studied regions, CGIs and TFBRs showed the most significant contribution of this non-processive (termed ‘region-based’) correlation, which persisted past 1 kbp. In contrast, SINE and LINE showed the least contribution, suggesting that CpG interdependence in these regions results nearly entirely from linear diffusion (processivity) of the enzyme. (iv) Further analysis of the region-based rate correlation indicates that much of the regional variation can be attributed to variation in three genomic/epigenomic features: chromatin accessibility, CpG density and bulk methylation.

More generally, we show how combining ‘top-down’ mathematical modelling (i.e. data-driven, using statistical inference) with a ‘bottom-up’ approach (i.e. using hypothesis-driven, or mechanistic models) can be used to glean kinetic insights from a measurement technique that affords genome-wide readout of the post-replication methylome over time [32]. A major new contribution of our paper is the development of a method for using data-derived rate correlations on genomic sites as a quantitative fingerprint of diffusive and non-diffusive enzyme kinetics. This method could be applied to other datasets in the future.

A number of experimental techniques have recently been developed, employing nucleoside-analogue labelling of replicating DNA, followed by isolation/immunoprecipitation and sequencing [21,39–48]. Our study shows that statistical correlations from such measurements have potential to yield quantitative insights into molecular mechanisms governing epigenetic inheritance, even when time resolution is coarse.

Diffusion of proteins along DNA has been an area of intense study, notably in the context of how transcription factors (TFs) locate target sites (reviewed in [49,50]). Direct measurement of linear diffusion of proteins along DNA *in vivo* has been achieved by single-molecule tracking [51,52]. Our study shows that, for enzymes with ubiquitous target sites on DNA, correlations from temporal sequencing can also yield quantitative details on protein diffusion along DNA within cells (albeit indirectly), without recourse to labelling and microscopy.

### 3.2. Processivity of DNMT1

While a number of studies have discovered evidence of DNMT1 processivity previously in *in vitro* systems [22–24,28,53,54], our study is the first to our knowledge to uncover a quantifiable signature of methyltransferase processivity in genome-wide, mammalian cell-based measurements. Thus our findings shed new light on enzymatic processivity within the context of maintenance methylation *in vivo*. Our findings are consistent with a picture wherein DNMT1 performs processive catalysis regardless of genomic context and in a quantitatively consistent manner (i.e. with relatively uniform lengths of processive steps between CpG targets, genome-wide). Our analysis does not afford direct estimation of linear coefficient *D*, but rather the ratio *D*/*k*_off_. We are not aware of any existing quantitative estimates or measurements of DNMT1 linear diffusion coefficient *in vivo*. If we assume that DNMT1 has *D* within similar range to other measured DNA-binding proteins (of order 10^5^ to 10^7^ bp^2^ s^−1^ [50,55,56]), our results would indicate average residence times of roughly 10^−4^ to 10^−1^ s for the enzyme when it is non-specifically bound to non-CpG sites, en route to catalytic sites.

Previous *in vitro* estimates of the length of processive runs of DNMT1 varied widely. For example, Vilkaitis *et al.* reported processive runs as long as 520 bp [23], while Goyal *et al.* reported processive runs of over 6000 bp [28]. Our estimates based on the Repli-BS dataset are much shorter than these, with the average length of a processive run being about 36 basepairs for nearby CpGs. One possible explanation for the discrepancy is that our estimate is from experiments in cells, where the *in vivo* chromatin environment, replication machinery and full complement of DNA-binding proteins are present. These could limit free diffusion of DNMT1 along DNA. TFs are also thought to search for targets partly by one-dimensional diffusion along DNA, and the effect of crowding has been considered [50]. While *in vitro* estimates for one-dimensional sliding lengths of various DNA-binding proteins are as high as 20 kb [55], *in vivo* sliding length of the lac repressor was found to be short, at 45 bp [51]. How the chromatin environment affects the motion of eukaryotic DNA-binding proteins is still poorly understood [57]. In particular, the question of how methyltransferase processivity is affected by the crowded environment of the cell warrants further study.

Our analysis of the exponential contribution to rate correlation cannot be directly or certainly attributed to the action of DNMT1 alone. For example, PCNA plays a role in recruitment of DNMT1 to replication foci [58], and this protein also can diffuse linearly along DNA [56] (although DNMT1 processivity does not rely on PCNA [23]). We cannot exclude the possibility that some mechanisms other than intrinsic DNMT1 diffusion along DNA give rise to the observed rate correlations, although this interpretation is consistent with the uniform lengthscale observed across different chromatin environments.

### 3.3. Non-processive CpG coupling

We find that additional mechanisms affect post-replication remethylation rate correlation where the local genomic/chromatin landscape allows it. We find this non-processive (i.e. non-exponential) source of rate correlation to be most prevalent where chromatin is locally open, CpGs are dense, and the background methylation is relatively low. Although our study does not attempt to define the mechanistic basis of the non-processive component of the rate correlation, various mechanisms can be proposed based on our observations and on previous literature. For example, any mechanism whereby DNMT1 reaches its CpG target through cooperative interactions with other molecules could be speculated to have kinetics dependent on the local genomic and epigenomic context. Neighbour rate correlations could then be sensitive to local context and have lengthscales determined by the cooperative molecular interactions, rather than being solely dependent on linear inter-CpG genomic distances, in contrast with the processive mechanism. For example, recruitment of DNMT1 to replicating DNA by UHRF1 [25,26] probably results in context-dependent kinetics, since UHRF1 targeting is dependent on both histone state and the presence of hemimethylated CpGs [59]. A recent finding of monoubiquinated histone H3 helping recruit DNMT1 to DNA stretches with multiple, but not one, hemimethylated CpGs [20] supports the idea that UHRF1 helps direct DNMT1 to CpG-dense regions and is consistent with our observation of higher rate correlation in CpG-dense regions. Our findings may also be consistent with a nucleation model, in which the initial binding of DNMT1 to replicating DNA occurs on nucleosomes, directed by UHRF1, after which DNMT1 reaches nearby CpGs processively [60]. If the initial binding events of separate DNMT1s on nearby nucleosomes are correlated, such correlation would contribute on a lengthscale of the order of hundreds of basepairs, while shorter-lengthscale correlation would be introduced through processivity.

In addition to UHRF1-mediated mechanisms, additional factors are likely to play a role in the non-exponential correlation we observe. First, DNA is not one-dimensional; DNMT1 could reach nearby CpGs by facilitated diffusion (combining one-dimensional diffusion along DNA with three-dimensional diffusion in the nucleosol to nearby sites) [50] or by intersegmental transfer, similar to TFs [49]. Our processive model assumes a one-dimensional substrate, but our results hint at sensitivity of maintenance kinetics to three-dimensional DNA structure in the weak appearance of peaks consistent with nucleosome spacing (figure 1). Finally, transient binding of post-replication DNA by TFs could introduce correlation into maintenance methylation kinetics, as TFs could transiently block access to CpGs by DNMT1 and thus delay remethylation. Such a mechanism could explain why we observe the most pronounced region-based correlation in TFBRs.

### 3.4. Relationship between WGBS and rate correlation

Our study reveals a significant difference between the local correlation of methylation state versus rate. We interpret this result as being due to the different information content of the two: WGBS experiments capture reads that largely reflect the stable methylation landscape (though still partially influenced by replication-associated temporal variability [32]), while the Repli-BS-derived methylation rates reflect the transient dynamic processes occurring post-replication. Of note, there appears to be some relationship between the two, as we observed similar trends in region specificity in the methylation state and rate (specifically in the region-specific, or non-processive, contribution to correlation). We furthermore note that, when we restrict the analysis to common sites (thus retaining CpGs with intermediate to high methylation levels, as rates are only inferable for these sites), the similarity between rate and state correlation is increased (i.e. as in figure 3 versus figure 1, where the decay of bulk state correlation in SINE is nearly as rapid as the decay of rate correlation). This suggests partial cross-talk between the transient post-replication methylation events (including processivity) and the stable methylation landscape. Indeed, it was previously reported that CpG co-methylation decays within tens to hundreds of bp, with enzyme processivity proposed as its mechanistic origin [36].

However, our results also show that, in some regions (such as CGI), methylation state correlation is significantly longer lived than the processive lengthscale and significantly higher than the rate correlation in total, and so the relationship between state- and rate correlation is not directly clear. As our analysis is based only on replication-associated methylation reactions, it seems likely that our rate correlations are largely indicative of DNMT1-mediated maintenance methylation, which cannot by itself shape the methylation landscape. The stochastic models of this article focus only on sub-cell cycle timescales and methylation maintenance and thus assume that the methylation landscape (i.e. state correlation) is a static property dictated by the parent-strand landscape at the time of DNA replication. However, methylation models of multi-cycle dynamics suggest that differences in kinetic parameters, including maintenance kinetics as well as demethylating and de novo methylation, shape the global methylation landscape across mitotic cycles [19,35]. Our method for obtaining data-driven kinetic correlations could therefore be useful in the future to further improve these types of multi-cycle mathematical models.

### 3.5. Implications for stability of the methylation landscape

A number of mathematical modelling studies have provided support for the presence of interactions (also called ‘collaboration’) among CpGs in dynamic methylation processes, including in maintenance, de novo methylation and demethylation reactions [19,27,30,31]. These models, in which a CpG is in some way affected by the state of nearby CpGs, built upon the earlier, so-called standard model, wherein each CpG was considered to be independently targeted by methyltransferases [61]. Crucially, as the cited studies showed, these interactions provide the necessary nonlinearity to enable bistability in the dynamic system. That is, they enable the same family of ‘reader’ and ‘writer’ enzymes to simultaneously maintain distinct states of hyper- and hypo-methylation on groups of CpGs in different parts of the genome, thus mimicking observed methylation patterns. While these models tend to be phenomenological in nature (i.e. capturing dynamic phenomena without necessarily encoding detailed molecular mechanisms), processivity can be considered to be one type of molecular mechanism that contributes to inter-CpG interactions. Indeed, mathematical modelling also supports the idea that diffusive processivity enhances multi-generational stability of methylation patterns [28], just as does other mechanisms of CpG interaction [19]. It follows that interdependence of CpG methylation kinetics, as quantified by correlation in this article, has relevance to human ageing and disease, since instability of the methylation landscape has been linked to both [62,63].

The mathematical model of Haerter *et al*. predicted that local (nearest-neighbour) interactions of methylating reactions was sufficient to achieve stable propagation of methylation states over multiple generations, though longer-range interactions were required for demethylating reactions [19]. In the present study, we find that maintenance methylation occurs largely independently in regions that are CpG sparse and show low region-dependent correlation, such as LINE. A nearest-neighbour-only model is consistent with our findings in regions such as 5UTR and SINE, where the processive lengthscale is on the same order as the typical inter-CpG distance. In CGIs, the processive lengthscale is longer than the inter-CpG distance (median 10 bp in the analysed data), suggesting that CpGs in CGIs effectively interact beyond nearest neighbours. Strong coupling of methylation in CGIs is consistent with faster maintenance kinetics in CpG-dense regions, as has recently been reported [21]. TFBRs showed inter-CpG distances similar to the processive lengthscale; however, here processivity is compensated by weak but longer-range (region-based) coupling (also evident in CGIs). These findings may predict enhanced stability of methylation in TFBRs and CGIs across mitotic cycles, although better understanding of the interplay of correlation lengthscales for de novo and demethylating reactions is needed.

### 3.6. Limitations of our study

Our study shows that novel experimental techniques that probe replication-associated dynamics genome-wide can yield surprisingly detailed dynamic insights despite the limited time resolution. However, the statistical inference approach is nevertheless limited. First, each site-specific inferred rate constant is the result of a fit of the data to a stochastic Poisson process; undoubtedly this is a simplistic model for dynamics that could potentially be temporally complex in reality, e.g. non-exponential or even non-monotonic. Thus, individual estimates can be error-prone for a number of reasons, from the inability of the simplistic model to capture complex dynamics, to the limited time resolution or sampling depth for a given site, or all of the above. Because of the inherent difficulty in characterizing dynamics in detail at any individual CpG genome-wide, we focused here on general features of the rate correlation functions that are robust across a given genomic region. In this way, the whole-genome nature of the data partially compensates for the sparse temporal resolution. We also ensured that our inference and analysis pipeline performed well on synthetic datasets, generated from simulations. Going forward, it may be possible to yield more detailed dynamic insights with deeper sampling and more fine-grained time resolution (as achieved in recent experiments [21]), which could enable further investigation into detailed, local kinetic correlation.

Our study was performed on data from a particular cell line (HUES 64). Cell-type-specific differences in human methylomes have been reported (e.g. [64]). The hESCs analysed here were found to have bimodal CpG landscapes similar to those of somatic cell types [65], though significant intermediate methylation is also present and has been attributed to proliferation [32]. Although our mathematical modelling suggests that the dominant short-range correlation lengthscale we observed relates to enzymatic properties and is thus probably not sensitive to cell-type-specific differences in methylation landscapes, further studies on other cell types will be needed to determine the generalizability of our findings.

While we focus our models around the enzyme kinetics of DNMT1 (for simplicity and because it is the dominant methyltransferase responsible for carrying out maintenance activity), we acknowledge that our rate correlations are probably impacted by the presence of other (de novo) methyltransferases, which have been known to associate with highly methylated CpG-dense location (i.e. CGIs) [66]. Although our mechanistic models rigorously incorporate one-dimensional diffusion, they lack the dynamic interplay of de novo, maintenance and demethylation reactions that has been studied in mathematical models previously [14,19]. Our approach could be applied to different cell lines in the future, e.g. with specific methyltransferases disrupted, to further disentangle the molecular basis of kinetic correlation.

Another limitation of the model is that it does not directly include the dynamics of DNA replication, nor account for the presence of multiple distinct origins of DNA replication in the genome. Replication timing of distinct loci could introduce kinetic correlations in DNA methylation that were not directly analysed in our study. However, it is possible that rate correlation reported here could be related to replication origins, as CGIs have been partly linked to replication origin activity [67] and show more pronounced region-specific correlation in our study.

## 4. Material and methods

### 4.1. Site-specific post-replication methylation kinetics inference

The post-replication methylation data (Repli-BS data) of human embryonic stem cells (HUES 64) were downloaded from GSE82045. In the Repli-BS experiments [32], cells were pulsed for 1 h with bromodeoxyuridine (BrdU). Then, bisulfite sequencing of BrdU-labelled DNA captured CpG methylation reads from DNA that was replicated during the pulse interval. The pulse-chase experiment captured methylation level of CpGs at timepoints 0, 1, 4 and 16 h post-pulse, thereby giving a genome-wide temporal readout of CpG methylation over 16 h post-replication.

The MLE procedure for inferring per-CpG post-replication methylation rates from Repli-BS data is described in detail in [34]. Briefly, the temporally distributed binary read-data (methylated-1 or unmethylated-0) at each CpG site is fitted by a Poisson process, with each site *i* characterized by two inferred constants, *k*_*i*_ and *f*_*i*_, which represent the rate at which methylation accumulates at the site over the course of the experiment, and the steady-state (or long-time) fraction of cells in the measurement set that exhibit methylation at site *i*, respectively. From here on, we refer to the inferred parameters *k*_*i*_ as the ‘post-replication remethylation rates’ or simply the ‘remethylation rates’. Details of the inference approach can be found in the electronic supplementary material (Extended methods). Note that, while the per-site inferences are obtained based on an analytical, independent Poisson process model, the inferred rate parameters can nevertheless be used to investigate more complex types of dynamics and inter-site dependencies through correlations that are observed among inferred parameters on nearby CpGs.

The ability to infer a remethylation rate for a given CpG site, and the uncertainty associated with that inferred rate, depends on the read-depth of the experimental data, which varies across sites and across timepoints. Details of uncertainty quantification can be found in the electronic supplementary material and in our previous study [34]. We estimated on average 30% error in any given estimate of rates *k*_*i*_. We validated our method by ensuring that ground-truth rate correlations (obtained from simulated data) could be accurately recovered by the MLE inference pipeline.

### 4.2. Annotations of genomic regions

The GRCh37/hg19 genome was used as the reference genome in this article. The region annotations for genes, promoters, exons, introns, 3’UTRs and 5’UTRs are downloaded from the UCSC Genes track in UCSC Table Browser, whereas the LINEs (long interspersed nuclear elements), SINEs and LTRs were extracted from RepeatMasker track. CGIs and enhancers were downloaded from CGI track and GeneHancer track, respectively. The promoters in this article were defined as regions 2000 bp upstream and 200 bp downstream of transcription start sites. Local CpG density for a site was defined as the number of neighbouring CpGs in a 500 bp window centred at that site.

The chromatin accessibility data were retrieved from ENCODE/OpenChrom (Duke University) H1 cell line. The regions of TF peaks or TFBR denoted in this article were acquired from ENCODE ChIP-seq clusters for 161 TFs in H1 cells. The whole genome bisulfite sequencing (WGBS) dataset used in this study was retrieved from GSM1112841.

### 4.3. Stochastic simulation

Region-specific simulations of single-CpG stochastic enzyme-kinetic models were carried out using two candidate mechanisms, the distributive model and the processive model (figure 4). The model reactions and associated rate parameters are graphically depicted in figure 4*b*,*c*. The distributive model was simulated using the Gillespie stochastic simulation algorithm [68]. To incorporate one-dimensional diffusion into the processive model, we used a first passage time kinetic Monte Carlo algorithm inspired by [69]. The methylation maintenance model and simulation method are based in part on our previous simulation studies [34]. In the present article, we refined our processive model and simulation algorithm to rigorously incorporate physics of one-dimensional diffusion (also called ‘sliding’) of proteins along DNA, with unbinding [50], while enabling simulation of large numbers of CpGs. Our analytical results on enzyme diffusion and simulation algorithm are presented in the electronic supplementary material (Extended methods), along with further details of the models. Parameter values are chosen to be in line with experimentally measured values for DNMT1 where possible [70] and also to match features of the Repli-BS data (see electronic supplementary material, table S1 for details).

Simulations are performed for stretches of *N* CpGs (*N* = 25 000−75 000). Simulations mimic two types of experimental data modalities: WGBS and Repli-BS. To simulate WGBS experiments, for a region of CpGs, the simulation is initialized at post-replication time = 0, and then read out at randomly sampled timepoints between 0 and 24 h later, to reflect the variable post-replication timings of bulk cells in WGBS experiments. Ten simulation replicates are combined to generate estimates of average per-site methylation levels. To mimic the Repli-BS experiment, the methylation status of CpGs in the simulation were read out at timepoints sampled from intervals matching pulse-chase experiments [32], including uncertainty with respect to true post-replication timing. That is, given the finite BrdU pulse-length, the 0 h experimental timepoint is assumed to correspond to *t* ∈ [0−1] hours post-replication, and the 1 h experimental timepoint corresponds to *t* ∈ [1−2] hours post-replication, etc. Therefore, timepoints of simulation readout were sampled from *t*_chase_ + *r* ∈ [0, 1] h, where experimental timepoints *t*_chase_ were 0, 1, 4, 16 h and *r* is a uniformly distributed random number. The number of simulations and readouts at each timepoint were chosen to mimic the distribution of experimental read-depths. The synthetic Repli-BS data were then processed with the same MLE procedure as the experimental data to infer per-CpG methylation rates. Simulations were used to validate our statistical inference procedure. We tested that ground-truth correlation functions produced by the two models could be recapitulated by the inference procedure.

## Data Availability

Data (including inferred methylation rates and computed correlation functions) and simulation codes are available in a github repository: Read-Lab-UCI/DNA-methylation-kinetics-correlation. The data are provided in electronic supplementary material [71].
